# Hyperspectral Imaging Detects Clitoral Vascular Issues in Gender-Affirming Surgery

**DOI:** 10.3390/diagnostics14121252

**Published:** 2024-06-13

**Authors:** Torsten Schulz, Hannes Köhler, Lukas Herbert Kohler, Stefan Langer, Rima Nuwayhid

**Affiliations:** 1 Department of Orthopaedic, Trauma and Plastic Surgery, University Hospital Leipzig, 04103 Leipzig, Germany; stefan.langer@medizin.uni-leipzig.de (S.L.); rima.nuwayhid@medizin.uni-leipzig.de (R.N.); 2 Innovation Center Computer Assisted Surgery (ICCAS), University of Leipzig, 04103 Leipzig, Germany; hannes.koehler@medizin.uni-leipzig.de; 3 LVATE GmbH, 80333 Munich, Germany; lukas.kohler@lvate.de

**Keywords:** hyperspectral imaging, image-guided surgery, vaginoplasty, gender-affirming surgery, intraoperative imaging

## Abstract

The aim of this study was to assess the efficacy of hyperspectral imaging (HSI) as an intraoperative perfusion imaging modality during gender affirmation surgery (GAS). The hypothesis posited that HSI could quantify perfusion to the clitoral complex, thereby enabling the prediction of either uneventful wound healing or the occurrence of necrosis. In this non-randomised prospective clinical study, we enrolled 30 patients who underwent GAS in the form of vaginoplasty with the preparation of a clitoral complex from 2020 to 2024 and compared patients’ characteristics as well as HSI data regarding clitoris necrosis. Individuals demonstrating uneventful wound healing pertaining to the clitoral complex were designated as Group A. Patients with complete necrosis of the neo-clitoris were assigned to Group B. Patient characteristics were collected and subsequently a comparative analysis carried out. No significant difference in patient characteristics was observed between the two groups. Necrosis occurred when both StO_2_ and NIR PI parameters fell below 40%. For the simultaneous occurrence of StO_2_ and NIR PI of 40% or less, a sensitivity of 92% and specificity of 72% was calculated. Intraoperatively, the onset of necrosis in the clitoral complex can be reliably predicted with the assistance of HSI.

## 1. Introduction

Spectral imaging combines conventional imaging and spectroscopy to obtain information about an object [1]. Several types of spectral imaging systems for analysing different types of biological materials have been developed [1]. In biomedical applications, they can be categorised into four different technical approaches, termed whiskbroom, pushbroom, staring and snapshot [1]. Whiskbroom and pushbroom modes use prisms, gratings or similar elements to split light, making them efficient, low-cost and effective at reducing scatter. Both approaches work with a broad wavelength range, allow for the selection of specific wavelengths to be analysed, offer high spectral resolution and have high data throughput. Pushbroom mode, in particular, can use a single line of light, which is useful for detecting specific spectral signatures in real time [2]. However, they come with drawbacks: the optical designs are complex and traditional scanning takes longer to collect data [1,3]. The staring mode offers quick data collection and can easily be paired with other visual systems like cameras or endoscopes. It also provides flexibility in selecting the optimal spectral range for different applications, making it versatile for detecting organs, tissues and cells. However, it has lower spectral resolution, lower transmission rates (35–60%) and is more expensive than whiskbroom and pushbroom modes. Further disadvantages are a low throughput and the need for sequential acquisition, especially in multifluorophore imaging [2]. Snapshot mode does not require scanning, making it fast and ideal for time-sensitive applications such as observing fast-moving molecules or dynamic biological processes. It is also suitable for mapping oxygen saturation in the retina using a fundus camera. Despite its speed, this mode is still in development and often relies on complex designs and intensive computational processing, with limitations in spatial and spectral resolution [1,4].

Despite the disadvantages of the pushbroom approach, spectral imaging has been a valuable tool in a variety of medical specialties for in vivo diagnostics [5]. HSI using the pushbroom approach can visualise and characterise different tissue components by capturing and processing a wide spectrum of wavelengths from visible light to the near-infrared range (400–1000 nm) [6]. HSI has improved the understanding of tissue perfusion and enabled the nuanced analysis of physiological and pathological conditions, facilitating early detection of disease, guiding surgical procedures and optimising therapeutic interventions. In particular, the identification and analysis of specific wavelength ranges have opened up new analytical possibilities in tumour detection [7], wound care [8], detecting ocular diseases [9], burn depth assessment [10] or assessing free flap perfusion [11]. In the surgical disciplines, HSI has gained great importance through its ability to enable intraoperative image-guided surgery [12]. HSI has been tested as intraoperative imaging for perfusion evaluation in various surgical specialties, including colorectal surgery, hepatopancreatic biliary surgery, upper gastrointestinal surgery, urology, plastic surgery and neurosurgery [12].

Moreover, HSI and artificial intelligence have been widely used for tumour diagnosis in digital pathology and can transform hyperspectral images into standard histologic images [13]. The methods were tested and evaluated by segmentation networks and pathologists on gastric cancer, lung adenocarcinoma, intrahepatic cholangiocarcinoma and colorectal cancer tissues. The experimental results show promising results for the automatic generation of fully annotated benchmark pathology datasets [13].

As defined by the DSM-5, gender dysphoria manifests as profound and enduring psychological distress resulting from incongruence between an individual’s gender identity and the sex assigned to them at birth [14]. In 2016, almost 1 million adults in the United States were identified as transgender, which equates to approximately 390 adults per 100,000 [15]. The incidence of gender-affirming surgery (GAS) has significantly increased in the past three decades. The rate rose from 0.16 to 0.42/100,000 for female-to-male patients and from 0.23 to 0.73/100,000 for male-to-female patients in Sweden [16]. The most significant increase occurred after 2000 [16]. A similar trend is also conceivable in other Western countries. GAS has emerged as a central component of comprehensive care and support for transgender people. These surgeries have seen remarkable advances in both medical techniques and social acceptance [17]. Vaginoplasty is a cornerstone in the field of GAS, offering transgender women the possibility of aligning their physical appearance with their gender identity. The primary objective for the patients is to establish a vagino-clitoral complex that is both functional and aesthetically pleasing [18]. One of the most important steps during surgery is the preparation of the clitoris, which maintains the ability to climax. To remodel the glans penis into a neo-clitoris, a reduction clitoroplasty is performed while preserving the penile nerves. The neurovascular bundle of the penis is carefully separated from the dorsal side of the penis [18]. The sensitivity test correlates with the medium-term ability to achieve orgasms [18]. A perfusion disorder resulting in the loss of the clitoral complex is a significant complication of this procedure and can restrict sexual function.

This study aimed to assess the diagnostical value of an HSI camera system using the pushbroom approach for intraoperative perfusion imaging during GAS. It was hypothesised that HSI could measure blood supply to the clitoral complex and predict uneventful wound healing or the development of necrosis.

## 2. Materials and Methods

### 2.1. Study Design

The study was designed as a non-randomised, prospective investigation. Patients who underwent GAS in the form of vaginoplasty in our clinic were included. Data acquisition took place from 22 April 2020 to 27 February 2024. HSI data were acquired intraoperatively following the suturing of the clitoral complex. The study’s primary endpoint was the occurrence of clitoral necrosis or the uneventful wound healing within seven postoperative days. Patients who met the WPATH criteria and provided written informed consent for GAS and data acquisition were included [19]. Excluded from the study were patients who declined to participate. Thirty patients met the inclusion criteria. Those who exhibited uneventful wound healing concerning the clitoral complex were categorised into Group A, while all other patients were allocated to Group B. Patient characteristics such as age, BMI, ASA status, and comorbidities were systematically collected and subsequently subjected to comparative analysis. The study received approval from the institutional ethics committee (reference number 258/20-ek, date of approval 24 June 2020). The study was carried out following the protocol and adhering to the moral, ethical, and scientific standards outlined in the Declaration of Helsinki in 1975 and its revision in 1983.

### 2.2. Surgical Technique

The surgical technique is an operative standard procedure consisting of the following steps: At first, the patient is placed in the supine position with abducted legs. The skin of the scrotum is excised in the shape of a shield. The preserved skin is reserved for potential augmentation of the neo-vaginal canal. Next, bilateral removal of the testicles and spermatic cord at the external inguinal ring is carried out. Ligation of the spermatic cord is performed twice using surgical sutures. The external inguinal ring is closed. A circular incision 1.0–1.5 cm proximal to the sulcus coronarius enables the dissection of the penile shaft. After placing a catheter, the urethra is separated from the corpora cavernosa, exposing them up to their crura. Ventral incisions are made in both corpora, with complete excision of the erectile tissue. The dorsal penile arteria, nerve and vein are divided from the dorsum of the corpus cavernosum accompanied by a strip of subjacent tunica. To ensure the complete removal of all erectile function, any remaining cavernosal tissue is sharply excised. Haemostasis is attained through the application of ligatures and bipolar coagulation. Shaping the glans, along with a reduction in size, creates a neo-clitoris. The remaining skin of the penile shaft is inverted to form a skin flap shaped like a tube. This inverted skin flap can be supplemented with a scrotal skin graft after thinning and retraction of hair roots to ensure full skin coverage of the neo-canal. Sharp dissection of the main pelvic ligament is performed. The neo-vaginal cavity is bluntly separated between the anterior and posterior layers. The dissection follows the Denonvillier’s fascia. A foam-based stent is sewn into a tube shape and then serves as a guiding structure for the inverted penile shaft and the skin graft within the newly created cavity. The neo-clitoris is placed above the urethra. After fixing the clitoris to the skin surface, looping the neurovascular bundle under the pubic skin ensues, secured with stitches on each side. Prior to insertion into the neo-vaginal cavity, the penile skin tube is treated with human fibrin. Gentle pressure application for 3 min enhances tissue adhesion. The urethra is shortened to the required length and two drainages are inserted into the neo-labia majora [17]. Subsequently, the soft tissues are closed layer by layer. The drains are sutured in place and continuous suction is applied. Finally, a compression bandage is applied over the genital area, consisting of gauze, pads and stretch bandage tapes. The first dressing change is performed 48 h postoperation. The urinary catheter remains in situ for 7 days. Prophylactically, pain therapy is administered according to the WHO pain relief ladder, anticoagulation with low-molecular-weight heparin and antibiotic therapy consisting of Ceftazidime and Metronidazole for 5 days. The foam tube is removed after 7 days to assess the integration of the penile inverted skin. After that, dilation of the neo-vagina is maintained by using dilators of different sizes for 3 × 30 min per day. After discharge, patients are regularly scheduled for wound and follow-up check-ups in our outpatient clinic. Typically, after 3 months, another correction of the vaginoplasty is performed, if necessary, including enlargement or reduction of the labia majora, correction of the urethral opening or the release of adhesions or scar tissues along the introitus to the neo-vagina.

### 2.3. HSI Imaging

The commercially available TIVITA^®^ Tissue camera (Diaspective Vision GmbH, Am Salzhaff-Pepelow, Germany) was used for data acquisition. This commercial imaging device consists of a mobile tabletop base, a tiltable compact hyperspectral detector and sensor with an integrated lightning unit and evaluation software (TIVITA^®^ Tissue Suite, version 1.6.0.1, 2019, Diaspective Vision GmbH, Am Salzhaff-Pepelow, Germany). The physical operation of the device has already been described in detail in various publications [5,6]. In short, the camera system is designed to evaluate different spectra (i.e., light of various frequencies) reemitted by the tissue, where the molecule-specific reemitted spectrum is generated from the light spectrum initially emitted for examination by the halogen spotlights.

The camera uses a pushbroom imaging spectrograph to collect data along one axis (y-axis) and has a high-quality infrared CMOS sensor (CMV2000-2E12M1PP, AMS SENSORS (CMOSIS), Antwerp, Belgium) for quick data recording. An internal motor moves parts of the spectrograph to capture images without moving the camera or the patient. The camera captures a “hyperspectral data cube” that includes two spatial dimensions (x and y) and one spectral dimension (λ). The CMOS sensor’s maximum resolution is 2048 × 1088 pixels. For calculations, the relevant area is reduced to 960 × 780 pixels, which corresponds to 500 wavelength points between 500 and 1000 nm. The TIVITA^®^ Tissue Suite software further reduces this to 480 × 100 pixels for each measurement. The system consists of six 20 W halogen light sources [5,6].

The calibration of the spectrograph in the HSI camera is carried out once by the manufacturer. During production, the wavelength is calibrated using krypton gas lamp peaks. As part of the maintenance of the system by trained personnel, a white balance is carried out if required to compensate for any variations in the light source. If the correct measuring distance is maintained (50 cm), which is clearly signalled by the HSI system, no further calibration is necessary before measurements. Preparation is therefore limited to starting the device, positioning the HSI camera over the measurement object using the integrated holding arm, switching off the ambient light and triggering the data acquisition. The system corrects for any optical distortions and sensor noise. The LabView-based software application controls the data collection and analysis. The software balances the images, calculates parameters and performs automated data analysis to extract parameters from the HSI data cube. The final output includes color-coded parameter values and an RGB colour image. For balancing and to ensure accurate measurements without being affected by time or temperature, certain effects are corrected by the internal software. The parameters computed include Oxygenation (StO_2_), Tissue Haemoglobin Index (THI), NIR Perfusion Index (NIR PI) and Tissue Water Index (TWI). Each is derived from specific regions of the spectrum [5,6].

Haemoglobin is key when analysing and distinguishing between its oxygenated and deoxygenated forms. To measure the oxygen saturation of the microcirculation, the spectrum is first filtered using a Gaussian window with a width of 6 nm. In a further step, the camera calculates the second derivative of the spectrum. This process removes fixed and straight effects, making the absorption bands clearer. Since the absorption and scattering characteristics of melanin are almost straight in the relevant spectral range, this procedure minimises their effect on the computed parameters. Two major spectral ranges show changes in oxygenated and deoxygenated haemoglobin. The primary range is from 500 to 650 nm, where the maximum of oxyhaemoglobin is seen. The secondary peak is from 700 to 815 nm, where the 756 nm peak of haemoglobin is observed in the derivative spectrum. As the saturation changes, the ratio of the two peaks changes [5,6]. This change is used to calculate the saturation:

$${StO}_{2}\;in\;\%=\frac{\frac{{min\;(A\prime \prime )}_{\left[570\ldots 590nm\right]}}{r_{1}}}{\frac{{min\;(A\prime \prime )}_{\left[570\ldots 590nm\right]}}{r_{1}}+\frac{{min\;(A\prime \prime )}_{\left[740\ldots 780nm\right]}}{r_{2}}}$$

To adjust the results to a reference, scaling factors *r*_1_ and *r*_2_ are used. These factors were obtained from measurements performed during the arm occlusion test. The MOXY monitor (a tissue oximeter from Fortiori Design LLC, Hutchinson, MN, USA) was used for these procedures. Because oxygenation reflects the relationship between saturated and unsaturated haemoglobin, it is presented as a percentage (“%”) [5,6]. The NIR Perfusion Index focuses on areas of the skin where light photo penetration is more significant. The isosbestic point at 797 nm divides the band into two parts with opposite absorptions. To determine the NIR Perfusion Index, first the average levels for the range between 655 and 735 nm and between 825 and 925 nm in each absorbance spectrum are found [5,6]. The NIR Perfusion Index is calculated as follows:

$$NIR\;PI\;in\;\%=\frac{\frac{{mean(A)}_{\left[825\ldots 925nm\right]}}{{mean(A)}_{\left[655\ldots 735nm\right]}}-s_{1}}{s_{2}-s_{1}}$$

The calibration parameters *s*_1_ and *s*_2_ are designed to scale the values to a region between 0 and 100. Since the chosen spectral region is in the NIR range where there is little absorbance, the emitted light penetrates deeper tissue layers (approximately 3–5 mm). The Tissue Haemoglobin Index (THI) represents the amount of haemoglobin in the microcirculation of the tissue. The spectral band between 530 and 590 nm was selected because the absorption of haemoglobin is dominant in this region. The second spectral region is located between 785 and 825 nm. These regions were chosen because the absorptions of oxygenated and deoxygenated haemoglobin are nearly identical (isosbestic), so that changes in oxygenation do not affect the THI [5,6].

$$THI\;in\;\%=\frac{\frac{{mean(A)}_{\left[785\ldots 825nm\right]}}{{mean(A)}_{\left[530\ldots 590nm\right]}}-s_{1}}{s_{2}-s_{1}}$$

The water absorption at 960 nm is clearly visible in the absorption spectra of normal human tissues, such as skin. The amount of water is calculated using the index. The procedure is the same as for the Tissue Haemoglobin Index (THI) and the NIR Perfusion Index, but using the 880–900 nm and 955–980 nm ranges [5,6].

$$TWI\;in\;\%=\frac{\frac{{mean(A)}_{\left[955\ldots 980nm\right]}}{{mean(A)}_{\left[880\ldots 900nm\right]}}-s_{1}}{s_{2}-s_{1}}$$

**Figure 1:**
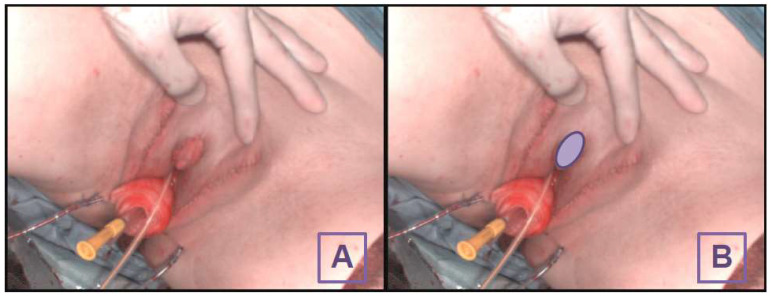
Intraoperative image of the clitoral hood without the ROI (**A**) and with the ROI (**B**).

This HSI system allows for detailed, accurate analysis of tissue characteristics without moving the camera or patient, making it efficient and reliable for medical imaging. The measurement process is contactless and non-invasive. The HSI measurement was conducted immediately after the insertion of the pedicled grafts during the operation. The evaluation process takes approximately 15 s. The study conducted further analysis by placing a region of interest (ROI) at the centre of the local flap/neo-clitoris (Figure 1). The four tissue parameters were assessed separately for each ROI. A clinical bedside examination was performed daily following the surgery up until the final clinical evaluation for signs of necrosis nine days postoperatively. Long-term follow-up was conducted during the second operative step of this two-part procedure three to six months later.

### 2.4. Statistics

Continuous data of both groups are delineated using the mean and standard deviation (SD), whereas qualitative data are expounded in terms of total numbers and percentages. A two-sided *t*-test was performed to detect differences in quantitative data. Qualitative data were tested using a Chi-Square test. *p*-values less than 0.05 were considered significant. The sensitivity and specificity were determined via analysis employing a confusion matrix. The data management was conducted using Excel (Microsoft Corporation 2018, Microsoft Excel), while the statistical tests were performed using SPSS (IBM Corp. Released 2022. IBM SPSS Statistics for Windows, Version 29.0. Armonk, NY, USA: IBM Corp). Furthermore, Python 3.6 with SciPy 1.2.3 and the Seaborn 0.11.2 library were used for statistical analysis of the HSI parameters and graphical data visualisation, respectively. Box plot charts were employed for visualisation and comparison of both groups. A coordinate system in the form of a scatter plot was used to visualise the cases in their respective groups.

## 3. Results

### 3.1. Study Cohort

The study cohort consisted of 30 individuals undergoing male-to-female gender confirmation surgery. Within the initial seven days postoperation, thirteen cases exhibited complete necrosis of the clitoral complex and were consequently classified into Group B. Cases characterised by uneventful wound healing of the neo-clitoris were designated as Group A. The comprehensive details of the cohort are delineated in Table 1.

The diagnosis of necrosis and assignment to Group A or B was confirmed on the day of patient discharge as well as during long-term follow-up three to six months later during the second surgical step of this two-staged procedure.

### 3.2. HSI Data

**Figure 2:**
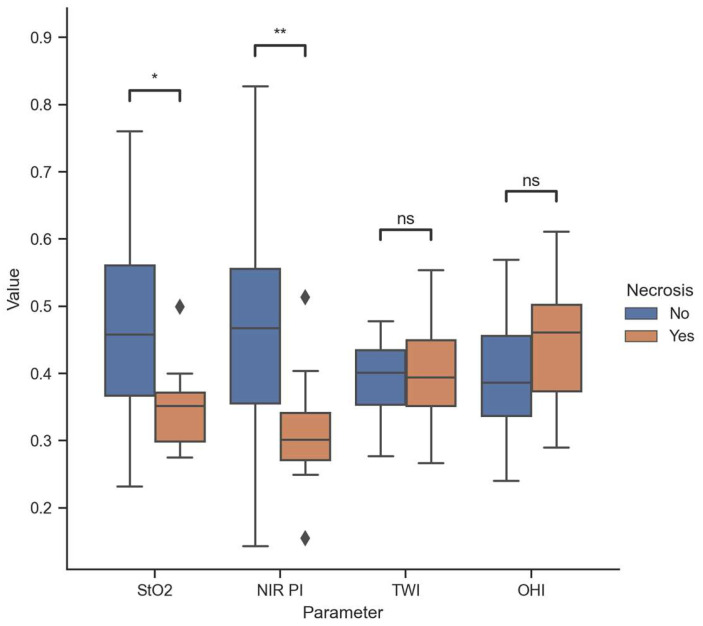
Descriptive data distribution of all perfusion parameters (range from 0 to 1). * 0.01 < *p* < 0.05; ** 0.001 < *p* < 0.01; ns 0.05 < *p* (not significant); diamonds indicate outliers.

The average measured StO2 and NIR PI in Group A were 46 ± 13% [23–76] and 46 ± 16 [14–82], respectively, compared to 35 ± 5% [27–49] and 31 ± 8 [15–51] in Group B, revealing significant differences with *p*-values of 0.008 and 0.02. Conversely, no sig-nificant differences were observed between the two groups in terms of TWI and OHI upon examination (*p*-values 0.98 and 0.28, respectively). Further details can be found in Figure 2.

### 3.3. Diagnostic Accuracy

**Figure 3:**
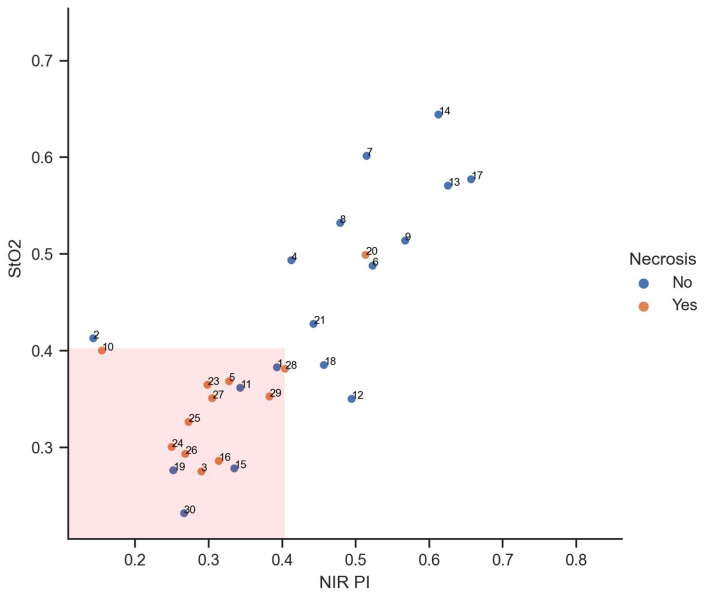
Indirect proportional relationship between the occurrence of necrosis and tissue oxygen saturation (range from 0 to 1 for both parameters).

Moreover, a clitoral complex with StO_2_ and NIR PI values below or equal to 40% indicated complete necrosis. In Figure 3, an inverse relationship between the occurrence of necrosis and tissue oxygen saturation is observed. As tissue oxygen saturation, represented by the parameters StO_2_ and NIR PI, increases, the occurrence of necrosis becomes less frequent, and vice versa. Despite a comparatively high tissue oxygen saturation, necrosis was observed in the subsequent clinical course solely in case number 20.

The diagnostic significance of breaching both threshold values was associated with a sensitivity of 92% and a specificity of 72%. The rate of false positive and false negative results stood at 28% and 8%, respectively. Further details are presented in Table 2.

The clinical assessments with subsequent categorisation into Groups A and B in the first postoperative days were consistent with the final assessments during long-term follow-up in all cases.

## 4. Discussion

In 1931, Abraham documented the initial account of a comprehensive staged genital reassignment procedure for a male-to-female transgender patient. This procedure involved lining the neo-vaginal cavity with a free split-skin graft [20]. In the context of vaginoplasty, the attainment of standardised techniques with consistently reproducible functional and aesthetic outcomes necessitated several decades of refinement. Notably, the construction of a sensitive neo-clitoris and the development of an aesthetically pleasing labial complex, commonly referred to as vulvoplasty, have emerged as increasingly significant considerations for both patients and surgeons in recent times [21]. A crucial surgical advancement in primary procedures involves the creation of a sensitive, well-vascularised neo-clitoris. Remarkably, this element did not become an integral component of surgical standards in sex-reassignment surgery until 1995 [22]. Since 1993, the majority of reports on male-to-female transgender GAS have consistently incorporated the creation of a neo-clitoris in their surgical descriptions. This procedure entails the dissection of a segment of the glans penis, including the dorsal neurovascular bundle, in continuity with the corpora cavernosa [21]. The meticulous subcutaneous positioning of the neurovascular bundle, ensuring torsion- and pressure-free placement, is paramount to prevent postoperative necrosis of the neo-clitoris. Approximately 80% of male-to-female patients who have undergone this procedure report experiencing erogenous sensibility of the neo-clitoris postoperatively [23,24,25]. This underscores the significance of this surgical step in not only achieving anatomical correctness but also preserving and enhancing sensory function for the overall well-being and satisfaction of the patients [21]. Today, the primary objective of GAS in transgender women is the creation of a perineogenital complex that embodies both feminine aesthetic and functional qualities [26]. A multidisciplinary team comprising mental health professionals, physicians, and surgeons is integral in facilitating the comprehensive care of transgender patients. GAS combined with cross-sex hormones and psychological therapy can improve the quality of life of transgender patients [27]. An enhancement in quality of life following GAS is well established [28]. In numerous studies, individuals undergoing GAS have reported notable enhancements in body image and an increased level of satisfaction with their overall lifestyle [29,30]. The majority of transgender individuals have communicated an improvement in their overall sexuality following GAS [31]. Additionally, more than half of the male-to-female transgender population in the study indicated experiencing a heightened orgasmic intensity post-surgery, surpassing their pre-transition experiences as males [24].

Each of the four technological approaches in spectral imaging technology demonstrates advantages and disadvantages [1]. Whiskbroom and pushbroom systems offer high resolution and low cost but are complex to set up. Staring mode is compact and easy to integrate with other instruments but is more expensive. Snapshot mode captures data quickly but has limited resolution [1]. Spectral imaging technology, by capturing images across a continuous range of wavelengths, offers insights into the spectral properties of various tissue components. This capability extends the applicability of spectral imaging to in vivo settings, thereby enabling non-invasive and real-time tissue assessment. A notable advantage of in vivo spectral imaging is its ability to detect both functional and structural pathological alterations in tissues, facilitating early disease diagnosis and grading without the need for tissue excision. Moreover, spectral imaging systems designed for in vivo detection can accommodate a broader spectrum of wavelengths, including the Near-Infrared (NIR) band, compared to those tailored for histological purposes. However, a significant constraint of spectral imaging in in vivo applications lies in its limited penetration depth due to the pronounced scattering of optical radiation within tissues. Consequently, tissues accessible via external surfaces (e.g., skin, retina, cervix, tongue, teeth, and tumour microvasculature) or endoscopic procedures (e.g., stomach and colon) are more amenable to in vivo spectral imaging [1]. Since its introduction for in vivo diagnostics, HSI using the pushbroom approach has offered both quantitative and qualitative assessments of tissue characteristics in a contact-free manner. Based on these characteristics, HSI using the pushbroom technology holds considerable promise as an intraoperative imaging technology [12]. The majority of preclinical studies are centred around HSI-based perfusion diagnostics in the operating room, validating this application across various organs. Furthermore, the quantification of tissue oxygenation, readily extractable from the hypercube, stands as a well-established and accessible piece of information that does not necessitate the involvement of complex machine learning algorithms. Presently, this application constitutes the most feasible use of HSI in clinical practice, a fact substantiated by the prevalence of numerous clinical studies spanning diverse surgical disciplines [12]. Several studies have substantiated the utility of HSI in the context of intraoperative image-guided surgery [32,33,34]. Our study constitutes a contribution, as it stands as the inaugural investigation into the perfusion conditions of the neo-clitoris within the framework of GAS. Regarding the analysis of tissue parameters provided by HSI, it is noteworthy that other studies arrived at comparable threshold values [12,35,36]. StO_2_ and NIR PI appear to play a pivotal role in assessing tissue perfusion, while OHI and TWI seem to assume a secondary role [36]. One reason for the false negative assessment of Patient Number 20 may be attributed, among other factors, to the intraoperative perfusion appearing unremarkable, only to subsequently experience diminished perfusion postoperatively, either due to haematoma formation or, for example, the dressing being applied too tightly intending to prevent postoperative bleeding. Causes for the false positive evaluation of Patients 1, 11, 15, 19, and 30 may be attributed to suboptimal examination conditions, such as excessive ambient light or an improper examination distance. Avoiding these unfavourable measurement conditions can affect the rate of false positive measurements.

The clitoral complex, as formed within the scope of this surgical procedure, anatomically resembles a pedicled flap. The arterial, venous and neural structures supplying the glans are meticulously dissected from the erectile tissue during the surgical intervention. Potential aetiologies for subsequent necrosis formation may encompass vascular compromise induced by electrocautery, torsion of the flap pedicle due to unfavourable rotation or the development of a hematoma within the pedicle region resulting in compression. In light of our findings, in instances where both parameters exhibit an intraoperative decline to levels below 40%, we advocate for a meticulous assessment of the clitoral complex, followed by a potential surgical revision to preclude the occurrence of pedicle torsion or hematoma accumulation beneath the flap.

To date, former studies have substantiated the utility of HSI in reconstructive surgery in identifying tissue ischemia. However, each study has been observational, lacking an intervention arm, and thus, no flap salvage rate has been delineated [11,35,36,37,38,39]. This is a crucial component in underscoring the benefits of HSI over clinical examination. All aforementioned clinical investigations constituted preliminary trials strategically devised to evaluate the applicability of this innovative technology within the procedural workflow of plastic surgery cases. HSI demonstrated proficiency in identifying ischemic flaps and quantifying flap perfusion. Nevertheless, comprehensive studies with meticulously designed protocols are imperative to elucidate the clinical significance of decision-making tools based on HSI for the assessment of flap perfusion. However, due to the necessary measurement distance of 50 cm and the vertical orientation of the camera relative to the examination area, examination in the dorsal lithotomy position was only feasible from the ventral aspect. Consequently, further examination of other organ segments intraoperatively, such as the posterior vaginal canal or the posterior commissure, was not possible with the HSI system. Moreover, persistent limitations exist across all intraoperative applications of HSI, hindering the widespread adoption of this technology within the operating room. Currently, the available HSI systems remain relatively cumbersome and are ill-suited for deployment in minimally invasive surgical procedures [12]. Despite the acceptability of the acquisition time in most modern devices, approximately 6 s, they fall short of delivering information in the form of high-quality videos. Consequently, these systems require a relatively immobile subject to mitigate the impact of motion artifacts [12]. Furthermore, HSI is characterised by a notable dependence on ambient illumination and restricted applicability in individuals with heightened pigmentation owing to prolonged light absorption. Further disadvantages of the commercial camera system tested here are a lack of automatic differentiation of tissue perfusion by the software and the possibility of continuous measurements. The camera system itself is rather bulky and the camera head can be difficult to adjust, while correct adjustment is vital to the measurements. In addition to this adjustment, there is a need to switch off external light sources in the operating room and ensure a constant power supply for a few minutes. Due to the described technology, reliable analysis is not possible in patients with darker skin tones.

Currently, fluorescence angiography (ICG) is gaining widespread recognition as a perfusion evaluation tool in gastrointestinal surgery [40]. However, the ongoing debate regarding its utility persists, as ICG does not offer quantification and its interpretation remains predominantly subjective. Consequently, various ICG quantification algorithms, which currently lack universal acceptance, have been implemented in clinical practice [41]. As of yet, a comprehensive resolution regarding the superior diagnostic efficacy between ICG and HSI in the context of intraoperative imaging remains elusive.

One notable strength of the study was the equitable distribution of patient characteristics across the various patient cohorts. The principal constraint inherent in this feasibility study pertained to the restricted number of compromised clitoral complexes, which represents the pertinent variable essential for evaluating monitoring techniques. Consequently, the objective of this clinical investigation was to evaluate intraoperative HSI with regard to its efficacy in the early detection of compromised perfusion in clitoral complexes. The study’s small sample size is a limitation. Additionally, the cause of necrosis formation in the clitoral complex could not be clearly determined. It remains uncertain whether it was due to arterial hypoperfusion, venous congestion, torsion of the flap pedicle or haematoma formation. To improve selectivity in future studies with larger sample sizes, categorising individual flap compromises may be beneficial. The advantages of HSI compared to clinical examination or improvement of the flap salvage rate remain uncertain and should be investigated within the scope of future studies. Furthermore, there are no data on the temporal progression of the clitoral complex HSI parameters due to the study design. As in various other domains of our society, artificial intelligence is making strides in HSI-based tissue differentiation and has the potential to assist in automated analyses to identify recurring ischemic patterns in the future [42,43,44]. Future studies should implement artificial intelligence algorithms for the detection of ischemic tissue conditions and to prevent following surgical complications. Also, a direct comparison between ICG and HSI in future studies holds the potential to further augment progress in surgical disciplines [40,45]. In addition, a comparison of potential costs and an economic comparison of HSI and ICG imaging systems should be addressed in further studies.

## 5. Conclusions

HSI amalgamates imaging, spectroscopy, and tissue oximetry into a dynamic, non-invasive and contactless modality. It yields valuable data for the surveillance of perfusion and oxygenation of clitoral complexes during GAS. This methodology exhibits the capacity to discern complications intraoperatively, notably in scenarios characterised by vascular impairments.

## Figures and Tables

**Table 1:** Preoperative findings, *N* = 30 patients.

Variables	Group A/*N* = 17	Group B/*N* = 13	*p*-Value
Age in years	34 ± 14	40 ± 16	0.34
BMI	26 ± 4	24 ± 5	0.16
ASA classification			
Grade I	13/76%	10/76%	0.97
Grade II	4/23%	3/23%	0.97
Grade III	0	0	-
Grade IV	0	0	-
Diagnosis			
Hypertension	1/5%	2/9%	0.39
Dyslipoproteinaemia	0	1/7%	0.24
Diabetes mellitus type II	1/5%	1/7%	0.84
Lung diseases	1/5%	0	0.37
Alcohol abuse	0	0	-
Smoking	5/29%	5/38%	0.6
Liver diseases	0	0	-
Medications			
Duration of hormone therapy in months	32 ± 18	39 ± 15	0.25
Cut-Stitch Time in minutes	226 ± 22	211 ± 17	0.18

None of the quantitative data series showed statistically significant differences between the groups.

**Table 2:** Diagnostical accuracy shown as a confusion matrix.

	Actual Necrosis		
Predicted necrosis	11 (true positive)	5 (false positive)	16
	1 (false negative)	13 (true negative)	14
	12	18	30
	$Sensitivity=\frac{11}{12}=92\%$	$Specificity=\frac{13}{18}=72\%$	

## Data Availability

The raw data supporting the conclusions of this article will be made available by the authors on reasonable request.
